# Biologic activity and safety of belimumab, a neutralizing anti-B-lymphocyte stimulator (BLyS) monoclonal antibody: a phase I trial in patients with systemic lupus erythematosus

**DOI:** 10.1186/ar2506

**Published:** 2008-09-11

**Authors:** Richard Furie, William Stohl, Ellen M Ginzler, Michael Becker, Nilamadhab Mishra, Winn Chatham, Joan T Merrill, Arthur Weinstein, W Joseph McCune, John Zhong, Wendy Cai, William Freimuth, the Belimumab Study Group

**Affiliations:** 1 Division of Rheumatology and Allergy-Clinical Immunology, North Shore Long Island Jewish Health System, Marcus Avenue, Lake Success, New York 11042, USA; 2 Division of Rheumatology and Immunology, University of Southern California Keck School of Medicine, Zonal Avenue, Los Angeles, California 90033, USA; 3 Division of Rheumatology, SUNY Downstate Medical Center, Clarkson Avenue, Brooklyn, New York 11203, USA; 4 Department of Medicine/Section of Rheumatology, The University of Chicago Hospitals, South Maryland Avenue, Chicago, Illinois 60637, USA; 5 Section of Rheumatology & Clinical Immunology, Wake Forest University Health Sciences, Medical Center Boulevard, Winston-Salem, North Carolina 27157, USA; 6 Division of Immunology and Rheumatology, University of Alabama at Birmingham, 510 20th Street, Birmingham, Alabama 35294, USA; 7 Department of Medicine, Clinical Pharmacology Research Program, Oklahoma Medical Research Foundation, 825 NE 13th Street, Oklahoma City, Oklahoma 73104, USA; 8 Department of Medicine, Section of Rheumatology, Washington Hospital Center, Irving Street NW, Washington, Distric of Columbia 20010, USA; 9 Division of Rheumatology, University of Michigan Health System, E Medical Center Drive, Taubman Center, Ann Arbor, Michigan 48109, USA; 10 Biostatistics, Human Genome Sciences, Inc., Shady Grove Road, Rockville, Maryland 20850, USA; 11 Pharmacology, Pharmacokinetics & Toxicology, Human Genome Sciences, Inc., Shady Grove Road, Rockville, Maryland 20850, USA; 12 Clinical Research, Human Genome Sciences, Inc., Shady Grove Road, Rockville, Maryland 20850, USA

## Abstract

**Introduction:**

This trial evaluated the safety, biologic activity, and pharmacokinetics of belimumab, a fully human monoclonal antibody that inhibits the biologic activity of the soluble form of the essential B-cell survival factor B-lymphocyte stimulator (BLyS) in patients with systemic lupus erythematosus (SLE).

**Methods:**

Seventy patients with mild-to-moderate SLE were enrolled in a phase I, double-blind, randomized study and treated with placebo (*n* = 13) or belimumab (*n* = 57) at four different doses (1.0, 4.0, 10, and 20 mg/kg) as a single infusion or two infusions 21 days apart. Patients were followed for 84 to 105 days to assess adverse events, pharmacokinetics, peripheral blood B-cell counts, serology, and SLE disease activity. Data from the study were summarized using descriptive statistics. χ^2 ^type tests were used to analyze discrete variables. The Kruskal-Wallis test, the Wilcoxon test, and the analysis of covariance were used to analyze the continuous variables, as appropriate. The analysis was performed on all randomized patients who received study agent.

**Results:**

The incidences of adverse events and laboratory abnormalities were similar among the belimumab and placebo groups. Belimumab pharmacokinetics were linear across the 1.0 to 20 mg/kg dose range. Long terminal elimination half-life (8.5 to 14.1 days), slow clearance (7 ml/day per kg), and small volume of distribution (69 to 112 ml/kg) were consistent with a fully human antibody. Significant reductions in median percentages of CD20^+ ^B cells were observed in patients treated with a single dose of belimumab versus placebo (day 42: *P *= 0.0042; and day 84: *P *= 0.0036) and in patients treated with two doses of belimumab versus placebo (day 105: *P *= 0.0305). SLE disease activity did not change after one or two doses of belimumab.

**Conclusions:**

Belimumab was well tolerated and reduced peripheral B-cell levels in SLE patients. These data support further studies of belimumab in autoimmune disorders.

**Trial Registration:**

NCT00657007 [clinicaltrials.gov].

## Introduction

Systemic lupus erythematosus (SLE) is typified by the production of autoantibodies, such as anti-double-stranded DNA (anti-dsDNA) antibodies and anti-nuclear antibodies (ANAs). Although the disease is characterized by the presence of autoreactive T lymphocytes, there is growing  evidence that B cells play a central role in the pathogenesis of SLE [1-3]. Hyperactive B cells may mediate disease by promoting the expansion of autoreactive CD4^+ ^T cells via antigen presentation [1-3]. The frequency of circulating plasma cells correlates with SLE disease activity and with the titer of anti-dsDNA autoantibodies [4]. Therefore, B-cell and plasma cell depletion may be an appropriate therapeutic approach in the treatment of SLE.

B-lymphocyte stimulator (BLyS) is a member of the tumor necrosis factor (TNF) ligand superfamily of cytokines that is expressed and secreted by monocytes, macrophages, dendritic cells, and granulocyte colony-stimulating factor activated neutrophils [5,6]. BLyS exists in both membrane-bound and soluble forms. The biologically active, soluble form of BLyS is enzymatically cleaved from the cell membrane and can bind to any of three receptors: TACI (transmembrane activator and calcium-modulator and cyclophilin ligand interactor) [7]; BCMA (B-cell Maturation Antigen) [8]; and BAFF-R (B-cell lymphocyte activating factor receptor)/BLyS receptor 3 [9,10], localized primarily on B lymphocytes. BLyS contributes to B-cell proliferation and differentiation, and it is important in immunoglobulin class switching [5,11]. Constitutive over-expression of BLyS in transgenic mice results in the development of an autoimmune-like disease that is characterized by hypergammaglobulinemia, autoantibody production, and glomerulonephritis [8,12,13]. In murine lupus, treatment with a BLyS antagonist significantly reduces the occurrence of proteinuria and prolongs survival [8,14]. Moreover, elevated BLyS blood levels have been found in some patients with SLE [15,16], and observational studies demonstrated that BLyS concentrations change over time in the majority of SLE patients [17,18]. Increases in BLyS levels correlated with increased disease activity and were predictive of future disease activity, suggesting that BLyS may be a biomarker for SLE [17].

Belimumab (LymphoStat-B; Human Genome Sciences, Inc., Rockville, MA, USA) is a recombinant, fully human, IgG_1λ _mAb that binds to soluble BLyS with high affinity. The antibody exerts its biologic activity by preventing the binding of BLyS to its receptors [19]. Belimumab potently inhibits BLyS-induced proliferation of B cells *in vitro *and prevents human BLyS-induced increases in splenic B-cell numbers and serum IgA titers in mice [19]. In cynomolgus monkeys treated with belimumab, reductions as great as 75% were observed in the number of lymphoid tissue and peripheral blood CD20^+ ^B cells and CD21^+ ^plasmacytoid cells [20]. Importantly, intravenous doses of up to 50 mg/kg delivered every 2 weeks over 6 months were well tolerated in cynomolgus monkeys. On discontinuation of belimumab in cynomolgus monkeys, the numbers of peripheral blood CD20^+ ^B cells recovered to normal levels within 3 to 5 months [20]. Because belimumab has the potential to provide therapeutic benefit in SLE patients, we conducted a phase I study of belimumab in SLE patients with stable, mild to moderate disease activity and demonstrated its safety, biologic activity, and pharmacokinetics.

## Materials and methods

### Patients

Patients aged 18 years or older with SLE, as defined by the American College of Rheumatology criteria [21], were enrolled in the trial. Eligible patients had stable SLE disease activity, as clinically judged by the principal investigator, for at least 2 months before screening and were either maintained with no medication or with a stable treatment regimen of low-dose (≤ 15 mg) prednisone, antimalarials, nonsteroidal anti-inflammatory drugs, methotrexate, azathioprine, or mycophenolate mofetil. Patients were required to have a history of measurable anti-dsDNA, anti-Smith, anti-ribonucleoprotein, anti-cardiolipin, anti-Sjögren's syndrome-A/Ro, or anti-Sjögren's syndrome-B/La autoantibodies. Patients with active lupus nephritis requiring hemodialysis, cyclophosphamide, or high-dose (>60 mg) prednisone, or who had received leflunomide, cyclosporin, intravenous gammaglobulin, or plasmapheresis within 6 months of screening were not eligible. Patients with active central nervous system lupus within 6 months of screening, a history of renal transplant, hypogammaglobulinemia or IgA deficiency, evidence of clinically significant non-SLE-related acute or chronic disease, or a history of any serious infection within 4 weeks of study entry were also excluded. Pregnant or nursing patients were ineligible for inclusion in the study, and adequate contraceptives were required in participating patients. The protocol was approved by each center's institutional review board, and all patients provided written informed consent.

### Study design and treatment

This was a phase I, multicenter (20 sites), randomized, double-blind, placebo-controlled, dose-escalation study of belimumab in patients with SLE. Patients received belimumab 1.0, 4.0, 10, or 20 mg/kg or placebo administered intravenously over at least 2 hours. Patients in cohorts 1 to 4 received a single dose of belimumab or placebo, whereas patients in cohorts 5 to 8 received two identical doses of belimumab or placebo 3 weeks apart.

Permission to escalate the dose was granted by the Human Genome Sciences Review Committee. The primary safety end-point for dose escalation was the incidence of grade 3 (severe) or 4 (life-threatening) adverse events (AEs). Dose escalation and initial dosing of the double-dose cohorts were not allowed if two or more patients in a cohort experienced a grade 3 or 4 AE, including hypogammaglobulinemia.

### Safety assessment

AEs were coded on the basis of the Medical Dictionary for Regulatory Activities terminology version 6.0 and were graded for severity according to the National Institutes of Health Division of Microbiology and Infectious Diseases Adult Toxicity Tables (Version May 2001). Adverse events and serious AEs were considered treatment emergent if they occurred within 84 days after the final dose of study agent (day 84 for single-dose cohorts and day 105 for double-dose cohorts). Hematology, clinical chemistry, and urinalysis panels were assessed on days 0, 2, 7, 14, 28, 42, 56, and 84 for patients in the single-dose cohorts, and on days 0, 2, 7, 14, 21, 23, 28, 35, 49, 63, 77, and 105 for patients in the double-dose cohorts. In a 4-week, preclinical monkey toxicology study, one (asymptomatic) animal treated with high dose belimumab (50 mg/kg) was found at terminal necropsy to have multiple splenic abscesses that might have existed before treatment. Therefore, abdominal computed tomography scans with oral contrast were performed randomly in half of the patients in each cohort at screening and 28 days after the last dose to evaluate the risk for abdominal infection. It should be noted that in a subsequent 26-week, multiple-dose (0 to 50 mg/kg) monkey toxicology study, belimumab was well tolerated and no abscesses or other toxicities were identified [20].

### Immunogenicity assessment

Blood samples were evaluated for anti-belimumab antibodies on day 0 before dosing and on days 14, 28, 56, and 84 for patients in single-dose cohorts; samples were obtained in the double-dose cohorts on days 0 and 21 before dosing and on days 14, 35, 49, 77, and 105. Samples were allowed to clot for 30 minutes at room temperature, centrifuged at 1,000 to 1,300 *g *for 10 to 15 minutes, and the serum was decanted and immediately frozen. The presence of anti-belimumab antibodies was determined using two screening ELISAs. The first assay was performed using the Fab portion of belimumab immobilized to a microtiter plate. Captured anti-belimumab antibodies were detected with horseradish peroxidase-conjugated goat anti-human IgG+IgA+IgM antibody and were quantitated by color conversion of tetramethylbenzidine. The second assay was performed using belimumab (whole antibody) immobilized to a microtiter plate. Captured anti-belimumab antibodies were detected with horseradish peroxidase-conjugated goat anti-human κ chain specific antibody, and they were quantitated by color conversion of tetramethylbenzidine. A serum sample was considered potentially positive for anti-belimumab if the mean A_450 _value of the postdose sample was at least twofold greater than the mean A_450 _value of the predose sample.

Samples that had tested positive in either screening assay were then examined in a neutralization assay. Predose and postdose samples were serially diluted and added to microplates coated with immobilized belimumab, followed by the addition of europium-labeled BLyS. Anti-belimumab antibody present in the sample would bind to the immobilized belimumab, and competitively inhibit binding of europium-labeled BLyS. Europium-labeled BLyS binding was quantitated by time-resolved fluorometric spectroscopy at 615 nm (excitation at 340 nm). A sample was considered to contain neutralizing anti-belimumab antibody if the mean postdose signal was statistically lower than the mean predose signal (*P *< 0.01, unpaired one-tailed *t*-test).

### Systemic lupus erythematosus disease activity assessment

Primary outcomes for clinical disease activity included the Safety of Estrogens in Lupus Erythematosus National Assessment (SELENA) Systemic Lupus Erythematosus Disease Activity Index (SLEDAI) [22], Flare Index [22,23], and the Physician's Global Disease Assessment (PGA). Proper use of these instruments was reviewed at the investigator's meeting. The PGA was based on a visual analog scale ranging from 0 (no disease activity) to 3 (severe disease activity). The Short Form-36 (SF-36; version 2) Health Survey, a self-administered survey, was incorporated to assess quality of life. All clinical disease activity measurements were assessed on days 0, 28, 56, and 84 for patients in the single-dose cohorts, and on days 0, 21, 49, 77, and 105 for patients in the double-dose cohorts.

### Pharmacokinetics

Serum concentrations of belimumab were determined by ELISA. BLyS-reactive belimumab was captured from diluted human serum onto BLyS-coated microtiter plates. Captured belimumab was detected using peroxidase-conjugated secondary mouse monoclonal anti-human IgG antibody. The lower limit of quantitation for this ELISA assay is 138.5 ng/mL of belimumab in 100% human serum.

### Biologic marker assessment

Biologic marker assessments included CD20^+ ^B cells and CD138^+ ^plasmacytoid cells, anti-dsDNA antibodies, ANAs, immunoglobulins (IgG, IgM, IgE, and IgA), and complement (C3 and C4). Anti-dsDNA antibodies and ANAs were measured by Farr assay (Specialty Laboratories, Santa Monica, CA, USA) and indirect fluorescent antibody assay (FOCUS Diagnostics, Herndon, VA, USA), respectively. Immunoglobulins, C3, and C4 were measured by nephelometry (FOCUS Diagnostics). Blood samples were drawn at screening (day 0) and on days 14, 28, 42, 56, and 84 for patients in single-dose cohorts, and at screening and on days 0, 14, 21, 35, 49, 63, 77, and 105 for patients in double-dose cohorts. Absolute counts of B cells and plasmacytoid cells were calculated on the basis of white blood cell counts multiplied by the percentage of lymphocytes and the percentage of cells staining for the CD20 and CD138 markers, respectively, as determined by fluorescence-activated flow cytometry. BLyS levels could not be measured because the presence of belimumab in the blood interfered with the detection of BLyS.

### Statistical methods

Data from the study were summarized using descriptive statistics. χ^2 ^type tests were used to analyze discrete variables. For continuous variables, the Kruskal-Wallis test was used to examine the difference across all treatment groups, and the Wilcoxon test was used to compare the differences between placebo and each of the belimumab-treated groups. An analysis of covariance was used to analyze the continuous variables if there was a significant difference in the variable at baseline. The analysis was performed on a modified intent-to-treat population, defined as the subset of all randomized patients who received study agent. All statistical analyses were performed using SAS (SAS Institute Inc., Cary, NC, USA), WinNonlin (Pharsight Corp., Mountain View, CA, USA), or R statistical packages.

## Results

### Patients

A total of 70 patients were enrolled in this study (Table 1). The majority (91%) of patients were female, and the median age was 38.5 years (range 22 to 80 years). Half of the patients were white, and 47% of patients were African American; all treatment groups included patients of Hispanic origin. The median duration of disease was 6.5 years (range 0.3 to 37.7 years). The majority of patients had disease manifestations that included ANA positivity (97%), immunologic disorder (89%), arthritis (87%), hematologic disorder (64%), or malar rash (56%) at the time of diagnosis. At baseline, 90% of the patients had ANA titers of 1:40 or greater, 60% were anti-dsDNA antibody positive (≥ 5 IU/ml) with some variability in median anti-dsDNA antibody levels (4.5 to 27.0 IU/ml) across dose groups, and an average SELENA SLEDAI score of 2.2 (range 0 to 8 points). There were no significant differences between treatment groups in terms of demographics, baseline disease duration, serology, or manifestations. Eighty per cent of patients were on an immunosuppressive agent, and there were no significant differences in the distribution of patients who were on mycophenolate, prednisone, or methotrexate (Table 2). However, there were more patients in the placebo group (38%) compared with the belimumab-treated group (12%) who were on azathioprine (*P *= 0.04). Thirty-six patients were randomly assigned to receive a single dose of study agent and 34 to receive two doses 21 days apart. All patients completed the study per protocol.

**Table 1:** Patient demographics and disease characteristics by treatment groups

Patient demographic or disease characteristic	Placebo (*n* = 13)	Belimumab				
		
		1.0 mg/kg (*n* = 15)	4.0 mg/kg (*n* = 14)	10 mg/kg (*n* = 14)	20 mg/kg (*n* = 14)	All active (*n* = 57)
Sex (*n* [%])						
Female	11 (85)	15 (100)	13 (93)	12 (86)	13 (93)	53 (93)
Male	2 (15)	0	1 (7)	2 (14)	1 (7)	4 (7)
Race (*n* [%])						
White	10 (77)	7 (47)	3 (21)	8 (57)	7 (50)	25 (44)
African American	3 (23)	8 (53)	11 (79)	5 (36)	6 (43)	30 (53)
Asian	0	0	0	1 (7)	1 (7)	2 (4)
Hispanic origin (*n* [%])	4 (31)	2 (13)	1 (7)	5 (36)	1 (7)	9 (16)
Age (years; median [range])	38 (30 to 58)	36 (22 to 56)	48.5 (23 to 62	37 (22 to 61)	38.5 (23 to 80)	39 (22 to 80)
Duration of SLE (years; median [range])	5.3 (0.4 to 15.3)	3.4 (0.4 to 13)	8.7 (0.4 to 37.7)	6.3 (1.8 to 20.8)	8.0 (0.3 to 29.4)	6.9 (0.3 to 37.7)
SELENA SLEDAI score (median [range])	4 (0 to 4)	2 (0 to 6)	0 (0 to 5)	2 (0 to 8)	2 (0 to 4)	2 (0 to 8)
ANA ≥1:40 at baseline (*n* [%])	12 (92)	13 (87)	14 (86)	13 (93)	13 (93)	53 (93)
Anti-dsDNA antibody (IU/ml; median [range])	9.5 (4.0 to 162.5)	6.0 (4.0 to 65.5)	4.5 (4.0 to 24.0)	27.0 (4.0 to 257.0)	5.0 (4.0 to 729.0)	6.5 (4.0 to 729.0)
Manifestations at the time of SLE diagnosis (*n* [%])						
Antinuclear antibody	13 (100)	14 (93)	14 (100)	14 (100)	13 (93)	55 (97)
Immunologic disorder	12 (92)	12 (80)	12 (86)	14 (100)	12 (86)	50 (88)
Arthritis	12 (92)	14 (93)	11 (79)	12 (86)	12 (86)	49 (86)
Hematologic disorder	7 (54)	14 (93)	9 (64)	7 (50)	8 (57)	38 (67)
Malar rash	6 (46)	8 (53)	5 (36)	12 (86)	8 (57)	33 (58)
Photosensitivity	7 (54)	7 (47)	6 (43)	9 (64)	8 (57)	30 (53)
Serositis	6 (46)	4 (27)	8 (57)	7 (50)	8 (57)	27 (47)
Oral ulcers	8 (62)	10 (67)	6 (43)	6 (43)	5 (36)	27 (47)
Renal disorder	4 (31)	2 (13)	4 (29)	6 (43)	4 (29)	16 (28)
Discoid rash	3 (23)	1 (7)	5 (36)	4 (29)	2 (14)	12 (21)
Neurologic disorder	0	3 (20)	1 (7)	1 (7)	1 (7)	6 (11)

ANA, antinuclear antibody; SLE, systemic lupus erythematosus; SELENA, Safety of Estrogens in Lupus Erythematosus National Assessment; SLEDAI, Systemic Lupus Erythematosus Disease Activity Index.

**Table 2:** Frequency of use of immunosuppressive agents during the study

Immunosuppressive drugs	Placebo (*n* = 13)	Belimumab				
		
		1.0 mg/kg (*n* = 15)	4.0 mg/kg (*n* = 14)	10 mg/kg (*n* = 14)	20 mg/kg (*n* = 14)	All active (*n* = 57)
Prednisone	9 (69)	14 (93)	9 (54)	10 (71)	8 (57)	41 (72)
Methotrexate	2 (15)	1 (7)	0	2 (14)	3 (21)	6 (11)
Azathioprine	5 (38)	2 (13)	2 (13)	2 (14)	1 (7)	7 (12)
Mycophenolate	0	1 (7)	1 (7)	1 (7)	5 (36)	8 (14)
None	3 (23)	1 (7)	5 (36)	3 (21)	3 (21)	12 (21)

### Safety

Overall, AEs were reported in 12 (92%) patients treated with placebo and 55 (97%) patients treated with belimumab (Table 3). The majority of AEs were mild to moderate in severity, and the incidence of AEs was similar in the placebo and belimumab (single and double dose) treatment groups. There was no increase in the incidence of infections in the belimumab groups (37% for all patients treated with active agent versus 62% for placebo), and only one infection (tinea pedis) was reported to be possibly related to study agent. The most common AEs in patients treated with belimumab were arthralgia (26%), headache (21%), rash (21%), diarrhea (18%), and nausea (18%). Although diarrhea and rash did not occur in the 13 patients who received placebo, these events were generally mild to moderate and were often felt to be unrelated to study drug. Furthermore, dose-dependent trends in those treated patients who developed diarrhea or rash were not observed. The most common AEs in patients treated with placebo were arthralgia (31%), nausea (31%), upper respiratory tract infection (15%), and joint swelling (15%). The frequency of AEs did not change with increasing doses of belimumab. Overall, there was no significant difference in the incidence of specific AEs between the belimumab groups and placebo.

**Table 3:** Incidence (three or more patients) of adverse events by dose: single-dose and double-dose cohorts combined

Adverse event	Placebo (*n* = 13)	Belimumab				
		
		1.0 mg/kg (*n* = 15)	4.0 mg/kg (*n* = 14)	10 mg/kg (*n* = 14)	20 mg/kg (*n* = 14)	All active (*n* = 57)
Arthralgia	4 (31)	3 (20)	2 (14)	7 (50)	3 (21)	15 (26)
Headache	1 (8)	3 (20)	3 (21)	4 (29)	2 (14)	12 (21)
Rash	0	4 (27)	2 (14)	2 (14)	4 (29)	12 (21)
Diarrhea	0	5 (33)	1 (7)	1 (7)	3 (21)	10 (18)
Nausea	4 (31)	2 (13)	3 (21)	2 (14)	3 (21)	10 (18)
Fatigue	0	1 (7)	2 (14)	3 (21)	1 (7)	7 (12)
Back pain	1 (8)	0	2 (14)	1 (7)	3 (21)	6 (11)
Joint swelling	2 (15)	0	1 (7)	0	4 (29)	5 (9)
Synovitis	1 (8)	2 (13)	0	3 (21)	0	5 (9)
Depression	0	3 (20)	0	0	0	3 (5)
Infections and infestations	8 (62)	4 (27)	8 (57)	4 (29)	5 (36)	21 (37)
Upper respiratory tract infection	2 (15)	0	3 (21)	1 (7)	3 (21)	7 (12)
Thrombocytopenia^a^	0	0	1 (7)	0	0	1 (2)
Pancreatitis^b^	0	0	0	0	1 (7)	1 (2)
Cellulitis staphylococcal^b^	0	0	0	1 (7)	0	1(2)
Sepsis^b^	1 (8)	0	0	0	0	0
Aspartate aminotransferase increased^b^	0	0	0	0	1 (7)	1 (2)
Blood creatinine increased^b^	0	0	0	0	1 (7)	1 (2)
Neutrophil count decreased^b^	0	0	0	2 (14)	0	2 (4)
Dehydration^b^	0	0	0	0	1 (7)	1 (2)
Pain in extremity^b^	0	0	1 (7)	0	0	1 (2)
Headache^b^	0	0	0	1 (7)	0	1 (2)
Sinus headache^b^	0	1 (7)	0	0	0	1 (2)
Angioneurotic edema^b^	0	0	1 (7)	0	0	1 (2)
Urticaria^b^	0	0	0	0	1 (7)	1 (2)

Values are expressed as n (%). ^a^Grade 4 potentially life-threatening adverse event. ^b^Grade 3 severe adverse event

The majority of AEs were considered not related or probably not related to study agent. In addition, there were no significant differences between placebo-treated and belimumab-treated groups in the incidence of grade 3 or 4 laboratory or hematologic toxicities (Table 4). Frequencies of hematologic or laboratory abnormalities did not vary with increasing dose or number of doses of belimumab. There were three patients (two in the 10 mg/kg double-dose cohort and one in the 4 mg/kg single-dose cohort) who developed grade 3 neutropenia (500 to 970/mm^3 ^absolute neutrophil count [ANC]) at one or two time points (days 14 or 63) during the study. The occurrences of neutropenia were not considered AEs because repeat complete blood counts within 1 week showed ANC to be returning to within the reference range or improving to mild or grade 1 severity. The patient (10 mg/kg double dose) with an ANC of 500/mm^3 ^recovered and was rechallenged on day 23 without recurrence of neutropenia. Two patients in the single-dose cohort (one 4 mg/kg and one 20 mg/kg) developed grade 1 or 2 neutropenia considered AEs. The patients receiving 4 mg/kg belimumab sustained grade 2 neutropenia (1,160 to 1,460/mm^3 ^ANC) on days 28 to 84. The patient receiving 20 mg/kg belimumab sustained grade 1 neutropenia (1,720 to 1,940/mm^3 ^ANC) on days 56 and 84.

**Table 4:** Summary of grade 3 and 4 laboratory and hematologic toxicities

Parameter	Grade	Placebo (*n* = 13)	Belimumab				
			
			1.0 mg/kg (*n* = 15)	4.0 mg/kg (*n* = 14)	10 mg/kg (*n* = 14)	20 mg/kg (*n* = 14)	All active (*n* = 57)
Activated partial thromboplastin time	3	0	0	1 (7)	0	1 (7)	2 (4)
Creatinine	3	0	0	0	0	1 (7)^a^	1 (2)
Hemoglobin	3	0	1 (7)	0	0	0	1 (2)
Hyperglycemia	3	0	1 (7)		0	0	1 (2)
Neutropenia	3	0	0	1 (7)^b^	2 (14)	0	3 (5)
Thrombocytopenia	4	0	0	1 (7)^b^	0	0	1 (2)
Proteinuria	3	0	0	0	1 (7)	1 (7)^a^	2 (4)
	4	0	0	0	0	1 (7)	1 (2)
Prothrombin time	3	3 (23)^c^	1 (7)^c^	0	0	0	1 (2)
	4	2 (15)^c^	0	0	0	0	0

Values are expressed as *n *(%). ^a^One patient had grade 3 serum creatinine and grade 3 proteinuria. ^b^One patient with grade 3 neutropenia and grade 4 thrombocytopenia after the first 10-mg/kg dose was rechallenged at grade 1 neutropenia, without further decline after the second dose. ^c^Four of six patients with grade 3 or 4 prothrombin time reported concomitant warfarin use, including the two patients with grade 4 prothrombin time.

Abdominal computed tomography scans with oral contrast revealed no evidence of infections or abdominal abscesses. Two patients developed a human anti-human antibody (HAHA) response. A single patient in the 20 mg/kg double-dose cohort (on concomitant mycophenolate mofetil and prednisone) developed detectable, non-neutralizing HAHA only on day 77. The other patient in the 1 mg/kg single dose cohort (on concomitant prednisone) had detectable neutralizing HAHA on days 14 to 56.

Ten patients (one placebo and nine belimumab) experienced a grade 3 (severe) AE, and one patient (belimumab) experienced a grade 4 (potentially life-threatening) AE of thrombocytopenia (Table 4), which was considered probably not related to the study agent. Most events were considered not related or probably not related to study agent. Grade 3 urticaria with chest pain was reported in one patient with a prior history of drug-induced urticaria during the administration of a 20 mg/kg single dose. The infusion was discontinued approximately 40 minutes after initiation, and the urticaria and chest pain resolved with two doses of intravenous diphenhydramine.

Six patients (one placebo and five belimumab) developed eight serious AEs, none of which were considered related to study agent. Patients in the belimumab single-dose cohorts reported diarrhea, dehydration, and sinus headache (one patient), staphylococcal cellulitis (one patient), and angioedema (one patient). Patients in the belimumab double-dose cohorts reported chest pain (one patient) and pancreatitis (one patient), whereas a patient in the placebo cohort developed sepsis (one patient). There was no significant difference in the incidence of severe or serious AEs between patients treated with placebo and those treated with belimumab. There were no deaths during the study.

### Pharmacokinetics

**Figure 1:**
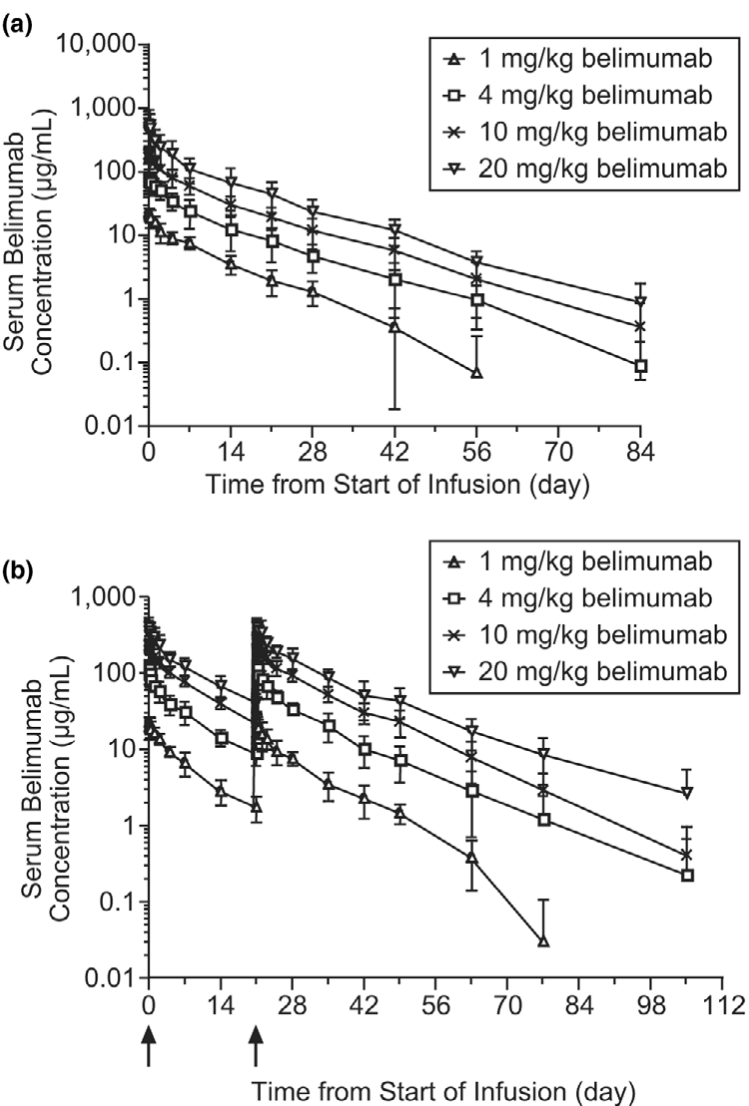
**Belimumab concentrations**. **(a) **Concentrations in the single-dose cohort. **(b) **Concentrations in the double-dose cohort. Arrows indicate time of belimumab administration. Values are expressed as mean ± standard deviation.

Following intravenous administration, serum belimumab concentrations declined in a bi-exponential manner, with a mean distribution phase half-life of 1.0 to 2.2 days and a mean terminal elimination half-life of 8.5 to 14.1 days (Table 5 and Figure 1). Belimumab was distributed to tissues with a mean steady-state volume of distribution ranging from 69 to 112 ml/kg, representing approximately twice the mean initial volume of distribution, which ranged from 40 to 57 ml/kg. The mean clearance after a single intravenous dose was approximately 7 ml/day per kg for all four cohorts, which is much less than the glomerular filtration rate, indicating that renal clearance is not a major component of belimumab clearance. Drug accumulation for maximum serum drug concentration averaged 9% when two doses of 4.0, 10, or 20 mg/kg were administered 21 days apart, which was as expected on the basis of the mean terminal elimination half-life of 9.6 to 14.1 days for those cohorts. There were no significant differences in pharmacokinetic parameters between single-dose and double-dose cohorts. Concomitant use of immunosuppressants, hydroxychloroquine, and/or prednisone during the study had no significant effects on belimumab pharmacokinetics (data not shown). Overall, belimumab pharmacokinetics were linear across the 1.0 to 20 mg/kg dose range, except in the two patients who developed anti-belimumab antibody responses. In these patients, the observed belimumab serum concentrations were 2-fold to 3.5-fold lower than the predicted values at the time points when anti-belimumab antibodies were detected.

**Table 5:** Pharmacokinetics parameters by dose levels following single and double doses of belimumab

Pharmacokinetic parameter (mean ± SD)	Belimumab dose and number of patients per cohort							
	
	Cohort 1 (1.0 mg/kg; *n* = 7)^a^	Cohort 2 (4.0 mg/kg; *n* = 7)	Cohort 3 (10 mg/kg; *n* = 7)	Cohort 4 (20 mg/kg; *n* = 6)^b^	Cohort 5 (1.0 mg/kg; *n* = 6)	Cohort 6 (4.0 mg/kg; *n* = 7)	Cohort 7 (10 mg/kg; *n* = 7)	Cohort 8 (20 mg/kg; *n* = 6)
C_max _(μg/ml)	22.3 ± 4.2	81.2 ± 24.6	192.4 ± 34.9	523.9 ± 293.7	20.6 ± 3.0	105.4 ± 28.0	240.7 ± 41.7	368.1 ± 93.5
C_max_/dose (kg/ml)	0.0223 ± 0.0042	0.0203 ± 0.0061	0.0192 ± 0.0035	0.0262 ± 0.0147	0.0206 ± 0.0030	0.0264 ± 0.0070	0.0241 ± 0.0042	0.0184 ± 0.0047
AUC_0-∞ _(day·μg/ml)	156 ± 46	629 ± 258	1,510 ± 315	3,384 ± 1,424	148 ± 30	729 ± 145	1,849 ± 355	3,221 ± 781
AUC_0-∞ _/dose (day· kg/ml)	0.1561 ± 0.0456	0.1572 ± 0.0646	0.1510 ± 0.0315	0.1692 ± 0.0712	0.1477 ± 0.0301	0.1822 ± 0.0363	0.1849 ± 0.0355	0.1611 ± 0.0391
t_1/2,α _(day)	0.96 ± 0.61	1.49 ± 0.76	1.84 ± 0.89	1.27 ± 0.43	1.87 ± 0.99	1.23 ± 0.65	1.03 ± 0.48	2.21 ± 1.84
t_1/2,β _(day)	8.46 ± 2.21	9.88 ± 2.18	10.63 ± 2.89	11.34 ± 3.02	9.67 ± 1.33	9.91 ± 2.99	9.64 ± 2.20	14.13 ± 5.31
V_1 _(ml/kg)	44.90 ± 7.12	52.69 ± 18.59	52.91 ± 10.20	53.17 ± 40.89	48.95 ± 8.26	39.61 ± 11.00	41.83 ± 7.63	56.60 ± 15.02
V_ss _(ml/kg)	73.29 ± 13.64	82.33 ± 22.31	86.30 ± 16.77	111.67 ± 95.72	76.45 ± 19.64	69.82 ± 22.72	69.21 ± 13.59	102.11 ± 30.40
CL (ml/day per kg)	7.15 ± 3.18	7.20 ± 2.48	6.90 ± 1.57	7.33 ± 4.38	7.00 ± 1.38	5.68 ± 1.11	5.57 ± 1.02	6.52 ± 1.54
MRT (day)	11.13 ± 3.08	12.18 ± 3.22	13.03 ± 3.59	14.01 ± 4.17	10.97 ± 1.86	12.47 ± 4.07	12.65 ± 2.66	16.06 ± 4.15

Belimumab was given as a 2-hour infusion. Cohorts 1 to 4 received single doses of belimumab. Cohorts 5 to 8 received two doses of belimumab 21 days apart. In the double dose cohorts, patients who missed doses or displayed positive immunogenicity were excluded. ^a^One patient in Cohort 1 was anti-belimumab antibody positive on days 14, 28, 56, and 84, and the data were, therefore, excluded from the mean calculation. ^b^One patient in Cohort 4 did not receive a full dose secondary to urticarial reaction, and serum concentration data from this patient were excluded from the PK analysis. AUC_0-∞_, area under the serum drug concentration-time curve from time 0 to infinite time; AUC_0-∞ _/dose, dose-normalized AUC_0-∞ _; CL, clearance; C_max_, maximum serum drug concentration; C_max_/dose, dose-normalized C_max_; MRT, mean residence time; SD, standard deviation; t_1/2,α_, elimination half-life for the distribution phase; t_1/2,β_, elimination half-life for the terminal phase; V_1_, volume of distribution for the central compartment; V_ss_, volume of distribution at steady state.

### Biologic activity

**Figure 2:**
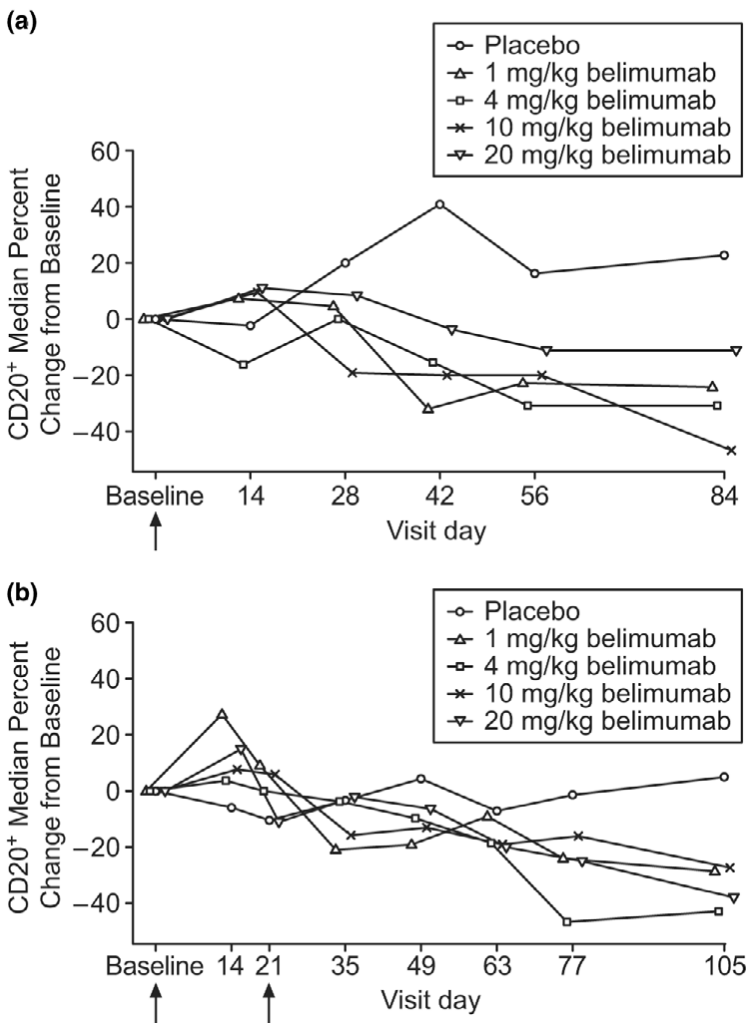
**Changes in CD20^+ ^B cells.** Median percentage change from baseline in CD20^+ ^B cells in **(a) **single-dose cohorts and **(b) **double-dose cohorts. Arrows indicate time of belimumab administration.

In general, the percentage reduction in CD20^+ ^B cells was greater in patients treated with belimumab than in those treated with placebo (Figure 2). The median CD20^+ ^B-cell count and percentage of lymphocytes at baseline were similar in the placebo (159 cells/ml and 13.5% [range 2% to 36%], respectively) and belimumab (176 cells/ml and 13.5% [range 2% to 51%], respectively) treatment groups. Baseline CD20^+ ^B-cell and CD138^+ ^plasmacytoid cell count data were not available for one patient in the 4.0 mg/kg single-dose belimumab cohort. Compared with placebo, a significantly greater reduction in median percentage of CD20^+ ^B cells was observed in the combined group of patients treated with either a single dose of belimumab (day 42: *P *= 0.0042; and day 84: *P *= 0.0036) or two doses of belimumab (day 105: *P *= 0.0305). The median reduction from baseline in CD20^+ ^B cells at day 84 for the single-dose cohorts ranged from 11% to 47%, whereas a 23% increase was observed in the placebo group. In the double-dose cohorts, the median reduction in CD20^+ ^B cells at day 105 ranged from 27% to 43%, whereas a 5% increase was observed in the placebo group. When patients with baseline values of 5% CD20^+ ^cells or greater were pooled across all cohorts (*n* = 65), the overall treatment effect was significant at 42, 56, and 84 days after the last dose (*P *< 0.01 for each). Patients on belimumab and mycophenolate mofetil (*n* = 8) had statistically significant differences in CD20^+ ^B cells at some time points compared with those (*n* = 49) on belimumab but not mycophenolate; however, the effects were not consistent throughout the study.

At baseline, the median CD138^+ ^plasmacytoid cell count and percentage of lymphocytes in the placebo group and combined group of patients treated with belimumab was 32 cells/ml and 2.5%, respectively. The median change from baseline in CD138^+ ^plasmacytoid cells at day 84 for the single-dose cohorts ranged from a 2.5% increase in the 1.0 mg/kg group to a 1.5% decrease in the 10 mg/kg group. In contrast, a 4.5% increase in CD138^+ ^plasmacytoid cells was observed in the placebo group. The overall treatment effect was statistically significant in favor of belimumab for the single-dose cohorts only (*P *= 0.0226).

**Figure 3:**
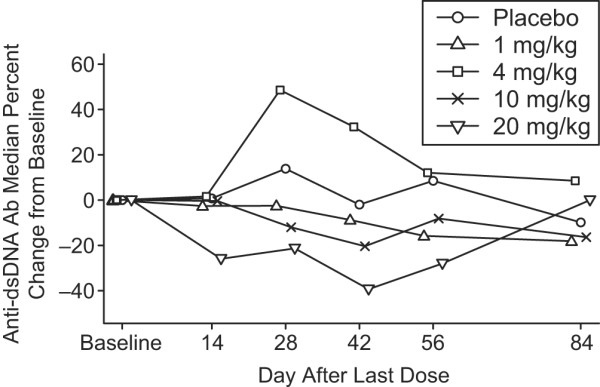
**Change in anti-dsDNA antibodies.** Mean percentage change from baseline in 31 patients whose anti-dsDNA antibody levels were 10 IU/ml or greater. dsDNA, double-stranded DNA.

Forty-four per cent of patients had elevations of anti-dsDNA antibody concentrations (normal <10 IU/ml) at baseline; the median baseline concentration of anti-dsDNA antibody was 22.0 IU/ml for patients treated with placebo and 27.5 IU/ml for patients treated with belimumab. Overall, the percentage change from baseline in anti-dsDNA antibody levels was not significantly different for the single-dose or double-dose cohorts compared with placebo. However, a subset analysis of 31 belimumab-treated patients with anti-dsDNA antibody levels 10 IU/ml or greater at baseline revealed significant changes from 28 to 56 days after the last dose across all cohorts (*P *< 0.05; Figure 3). Pair-wise comparison analyses confirmed that changes in anti-dsDNA antibodies in the 20 mg/kg dose group were statistically different from placebo at 28, 42, and 56 days after the last dose (*P *< 0.01 for each comparison). Of the three patients treated with belimumab who had exceedingly high anti-dsDNA antibody values (>200 IU/ml) at baseline, two had a decrease in anti-dsDNA antibody levels of more than 90% by the end of the study.

The percentage decrease in serum immunoglobulins tended to be greater in patients treated with belimumab (maximal median decrease over time for all doses combined was about 9% for IgG, about 11% for IgA, about 16% for IgM, and about 24% for IgE) compared with those treated with placebo; however, this trend did not achieve statistical significance. There were three patients (20 mg/kg double dose) whose screening and baseline IgG levels decreased from within the reference range (680 to 1,445 mg/dl) to below the lower limit of normal over 105 days (patient 1: 694 [baseline] to 527 [day 77] and 510 [day 105]; patient 2: 762 [baseline] to 651 [day 21] and 650 [day 105]; and patient 3: 809 [baseline] to 677 [day 105]). There were three patients (one receiving 4 mg/kg double dose, one receiving 1 mg/kg single dose, and one in placebo) whose screening and baseline IgM levels (38 to 45 mg/dl) decreased from within the normal reference range (33 to 248 mg/dl) to below (26 to 31 mg/dl) at different time points between days 14 and 77. None of the patients with normal screening and baseline IgA levels (70 to 407 mg/dl) dropped to below the normal range. Those patients (n = 13) with IgE levels above the reference range (>120 IU/ml) had a decline of approximately 16% at days 77 and 84. None of the reductions in immunoglobulin isotypes were considered to be an AE by the principal investigators. There were no significant changes in C4 or C3 across treatment groups.

### Clinical activity

**Figure 4:**
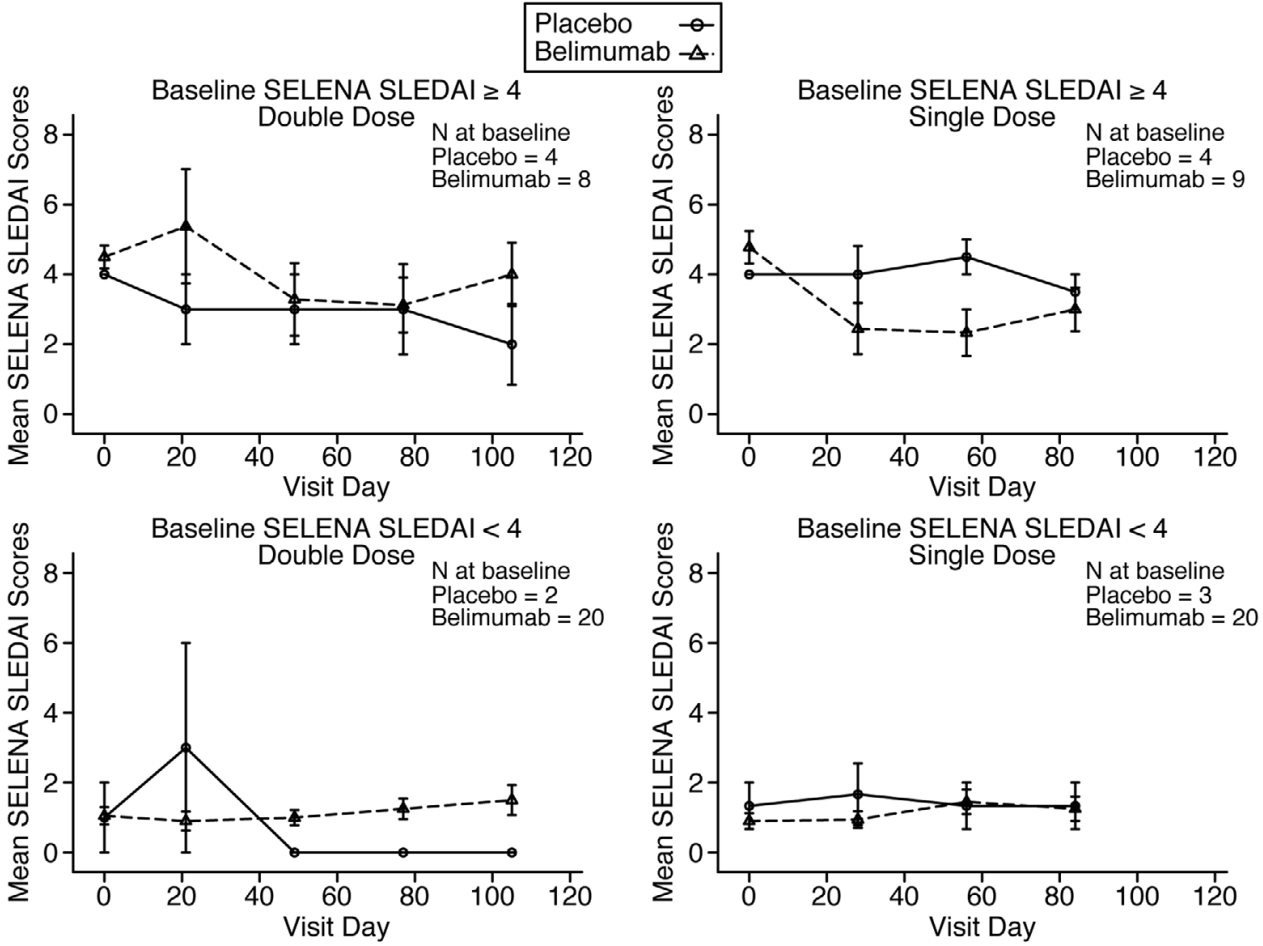
**SELENA SLEDAI scores.** The SELENA SLEDAI scores in the single-dose and double-dose cohorts over time are presented, stratified by baseline SELENA SLEDAI score (≥4 or <4). Values are expressed as mean ± standard error. SELENA, Safety of Estrogens in Lupus Erythematosus National Assessment; SLEDAI, Systemic Lupus Erythematosus Disease Activity Index.

The median baseline SELENA SLEDAI score for patients treated with placebo was 4 (range 0 to 4), with 33% of patients scoring 0. For patients treated with belimumab, the median baseline SELENA SLEDAI score was 2 (range 0 to 8), with 37% of patients scoring 0. Overall, there was a trend toward reduced SELENA SLEDAI scores in both belimumab and placebo groups. Changes in SELENA SLEDAI scores over time stratified according to the baseline SELENA SLEDAI score (≥ 4 or <4) for single- and double-doses are shown in Figure 4. Analysis revealed that the subgroup of patients with an SELENA SLEDAI score of 4 or greater had a median reduction of approximately 2 points 28 days after the last dose of belimumab; however, this trend reversed over the next two visits. The use of azathioprine or other immunosuppressive therapy at baseline or whether a patient was ANA positive (ANA ≥1:40) or negative at baseline did not significantly influence SELENA SLEDAI score responses in either belimumab or placebo groups (data not shown). Overall, there were no significant differences between belimumab and placebo treatment groups in SELENA SLEDAI scores or flare rates, as defined by the SLE Flare Index.

**Figure 5:**
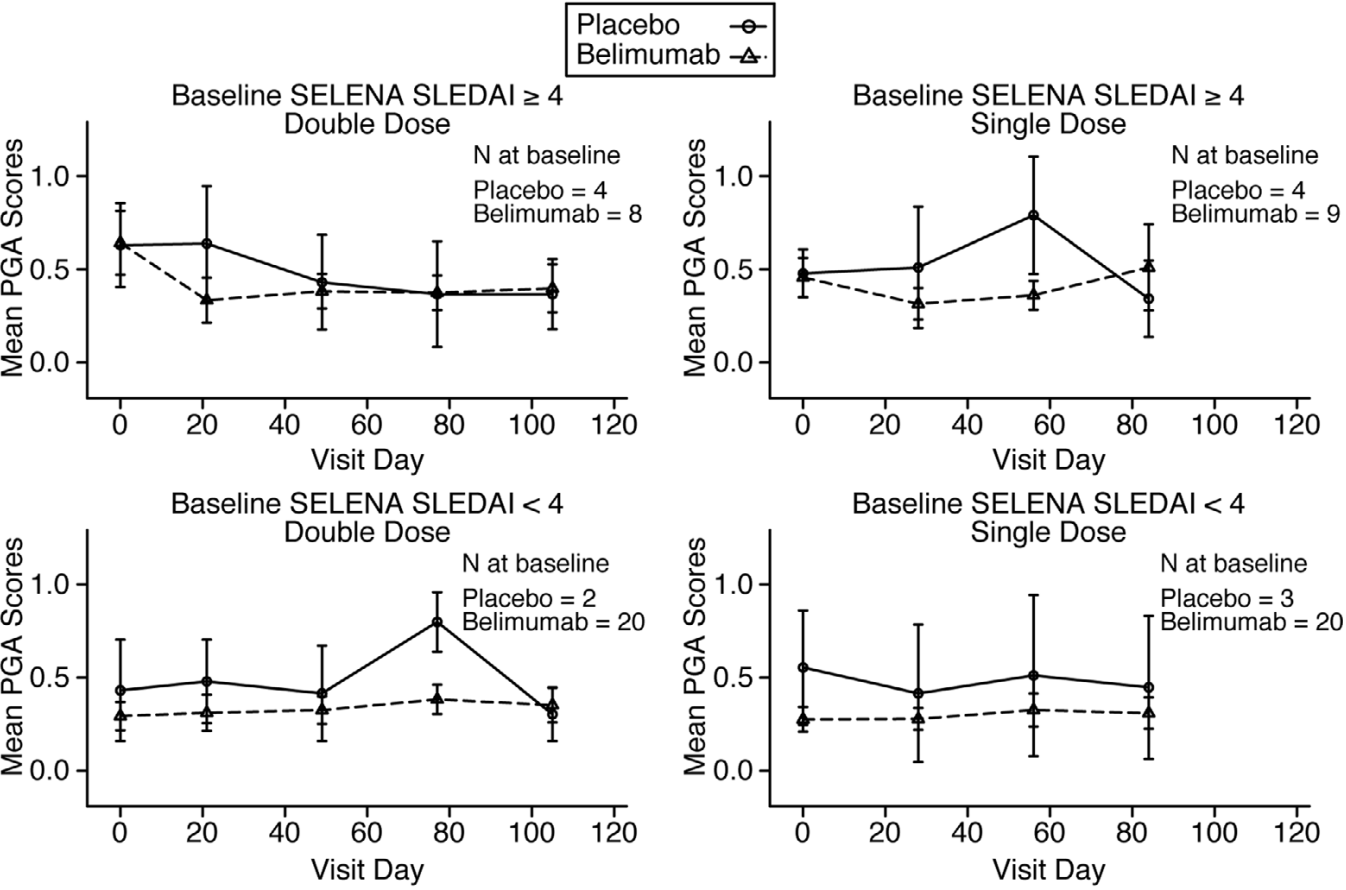
**PGA scores.** The PGA scores in the single-dose and double-dose cohorts over time are presented, stratified by baseline SELENA SLEDAI score (≥4 or <4). Values are expressed as mean ± standard error. SELENA, Safety of Estrogens in Lupus Erythematosus National Assessment; SLEDAI, Systemic Lupus Erythematosus Disease Activity Index; PGA, Physician's Global Disease Assessment.

**Figure 6:**
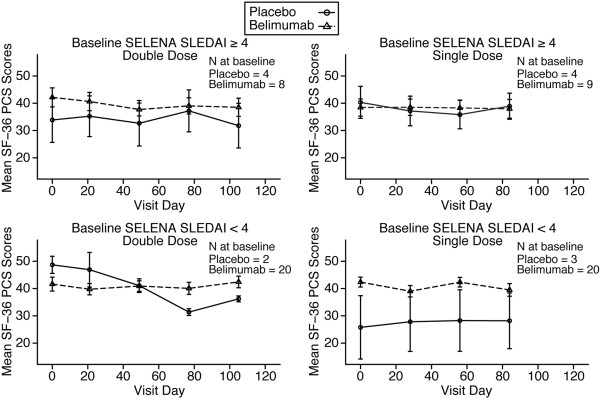
**SF-36 PCS scores.** The SF-36 PCS scores in single-dose and double-dose cohorts for over time are presented, stratified by baseline SS score (≥4 or <4). Values are expressed as mean ± standard error. PCS, Physical Component Score; SELENA, Safety of Estrogens in Lupus Erythematosus National Assessment; SF-36, 36-item Short Form; SLEDAI, Systemic Lupus Erythematosus Disease Activity Index.

The median baseline PGA scores, which ranged from 0.1 to 0.7 across all treatment groups in both cohorts, did not significantly change at any time point. Likewise, there were no significant differences among treatment groups in either absolute change or percentage change in any of the individual or combined scales of the SF-36 Health Survey. In addition, analysis of PGA and SF-36 Physical Component Score, stratified according to the baseline SELENA SLEDAI score (≥4 or <4) for single dose and double dose, did not reveal any significant changes in these parameters over 84 and 105 days of the study (Figures 5 and 6). The use of azathioprine or other immunosuppressive therapy at baseline or whether a patient was ANA positive (ANA ≥1:40) or negative at baseline did not significantly influence PGA or SF-36 responses in either belimumab or placebo groups (data not shown).

## Discussion

B cells play a prominent role in the pathogenesis of SLE, based on their ability to present antigen, secrete inflammatory cytokines, and produce autoantibodies. Therefore, B-cell depletion therapy for SLE has been an area of significant interest. Several treatment strategies that directly or indirectly affect B cells were recently investigated, including those that target BLyS, CD20, CD22 and CD154, and BLyS receptors [24,25].

CD40, a member of the TNF receptor superfamily, plays an important role in T-cell-mediated B-cell activation. Cross-linking of B-cell CD40 with CD154 (CD40 ligand), which is expressed on T cells, induces B-cell proliferation [26]. Concentrations of soluble CD154 have been reported to be significantly higher in patients with SLE than in control patients [27]. Early studies in SLE murine models suggested that blocking the interaction between CD40 and CD154 reduced nephritis and anti-dsDNA antibodies, leading to improved survival [28-30]. Furthermore, an anti-CD154 antibody, IDEC-131 (a humanized antibody that blocks CD40-CD154 interactions [31]), was well tolerated in a phase II study conducted in 85 patients with mild-to-moderate SLE [32]; however, it failed to meet the primary efficacy end-point, namely a reduction in SLEDAI score at 20 weeks after six infusions of IDEC-131 ranging from 2.5 to 10 mg/kg. A short course of another anti-CD154 antibody, BG9588, resulted in reductions in anti-dsDNA antibodies, increased C3 concentrations, and decreased hematuria in patients with proliferative lupus nephritis, suggesting that the drug has an immunomodulatory effect [33]. In a subset of this cohort, blockade of CD40 ligand with BG9588 markedly increased the frequency of IgG-producing B cells and IgG anti-DNA antibody producing B cells in the peripheral blood of treated patients. Development of this drug was suspended because of concerns about its prothrombotic effects [33].

Rituximab is a chimeric mouse-human mAb that is specific for CD20 and is currently approved for the treatment of patients with non-Hodgkin's B-cell lymphoma and patients with rheumatoid arthritis who have exhibited an inadequate response to at least one TNF antagonist [34]. Several studies have investigated rituximab in the treatment of SLE [35-38]. Recently, in two dose-escalation studies of 48 patients with SLE, rituximab therapy resulted in B-cell depletion and improved disease activity [35,37]. Rituximab generally appeared to be safe, although 12 out of 42 patients had documented human anti-chimeric antibody responses [37,38]. Two studies [37,39] have also demonstrated the safety of rituximab in the treatment of patients with lupus nephritis. Similarly, epratuzumab, a humanized anti-CD22 antibody, appeared to be safe in patients with SLE and resulted in immediate decreases in B-cell levels [40]. Antagonism of BLyS by TACI-Fc receptor or AMG 623 (Fc-peptide fusion protein [peptibody] with binding affinity for BLyS) has been evaluated in phase I SLE trials. Gradual reductions in CD20 B cells and immunoglobulin isotypes, particularly IgM, were observed [41,42].

Although anti-CD20 mAbs and BLyS antagonists both mediate B-cell depletion, recent studies suggest that B-cells targeted by anti-CD20 mAbs are not identical to those targeted by BLyS antagonists [1,5,6]. CD20 is expressed on most B-cell precursors in the bone marrow, immature B-cells, mature naive B-cells and memory B-cells. In contrast, BLyS receptors are not expressed on bone marrow B-cell precursors but are expressed on immature and mature B-cells, memory cells, and plasma cells. A recent study reported that a combination of anti-CD20 mAbs and BLyS antagonists achieved more effective B-cell depletion in a murine model than either agent alone [43]. Although treatment with agents such as rituximab results in B-cell depletion and improved disease activity [35,38], development of therapies that target other B-cell populations (for example, belimumab) may play a crucial role in enhancing B-cell depletion in patients with SLE. In addition, after treatment with rituximab in SLE patients, BLyS levels significantly increase after B-cell depletion until repopulation with B cells occurs [25,44].

In animal models, BLyS has been shown to be essential not only to the survival of B cells but also to the survival of plasma cells [45]. Belimumab inhibits soluble BLyS activity at subnanomolar concentrations in a murine model [19]. Belimumab, administered to monkeys at 5, 15, or 50 mg/kg doses every other week, affected peripheral B cells and lymphoid tissues as early as 3 months and 1 month, respectively [20]. Maximum reductions of up to 75% in CD20^+ ^B cells and CD21^+ ^plasmacytoid cells occurred at 13 to 26 weeks [20]. These effects were believed to be related to apoptosis induced by prolonged BLyS depletion and were reversible within 5 months of drug withdrawal.

The pharmacokinetic profile of one or two doses of belimumab was dose proportional and consistent with a fully human mAb. A half-life of 14 days supports dosing every 28 days in future long-term trials. A significantly greater reduction (up to 47% reduction) in the median percentage of CD20^+ ^B cells was observed in patients treated with one or two doses of belimumab compared with placebo. This is consistent with the ability of belimumab to inhibit BLyS biologic activity. Significant reductions in anti-dsDNA antibody were observed 28 to 56 days after the last dose in the subset of patients with levels of 10 IU/ml or greater at baseline. Immunoglobulin levels, particularly IgM and IgE, were reduced in some belimumab cohorts compared with placebo.

SELENA SLEDAI scores, PGA, SLE Flare Index, and SF-36 Physical Component Score did not significantly improve in this study population. The reasons for this relate to the limited number (one or two) of belimumab infusions, the short duration of the study, the relatively small numbers of patients, and the inclusion of patients with limited or no disease activity (33% had no disease activity at baseline). However, the demonstrated biologic activity suggests that long-term treatment may produce a clinical response and therapeutic benefit in patients with SLE. If the effects of belimumab in humans are comparable to those in monkeys, then we estimate that the depletion of CD20^+ ^B cells and CD21^+ ^plasmacytoid cells will be 50% to 80% within 3 to 6 months of continuous therapy. Significant reduction in B cells was observed in the lymphoid tissue of monkeys after 1 month of treatment but not observed in the peripheral blood until 2 months of continuous therapy [20]. Hence, clinical effects might be delayed until there is a sustained reduction in key B-cell populations. In a phase II SLE study of 449 SLE patients with baseline SELENA SLEDAI scores of 4 or greater (average SELENA SLEDAI score 9.6) treated with 1, 4, and 10 mg/kg belimumab or placebo plus standard of care SLE therapy, significant improvement in serologically active (ANA ≥1:80 and/or anti-dsDNA ≥30 IU/ml) [46] SLE patients in combined belimumab dose groups compared with placebo was seen at week 4 for PGA, week 12 for SF-36, and week 52 for SELENA SLEDAI score [47,48].

The results of this study demonstrate that treatment with one or two intravenous doses of belimumab is safe and well tolerated in patients with mild to moderate SLE. The AE profile of belimumab was comparable to that of placebo, and no serious AEs were deemed related to study agent. Only one patient experienced an infusion reaction, and this reaction responded to treatment with antihistamines. In addition, only one patient developed neutralizing antibodies to belimumab. Because belimumab is a fully human mAb, it is expected that patients are less likely to develop immune responses and hypersensitivity reactions.

## Conclusion

This study demonstrated that belimumab was biologically active *in vivo *and was safely administered to patients with SLE. These findings supported the initiation of phase II studies investigating the safety and clinical activity of belimumab in patients with SLE and rheumatoid arthritis.

## Abbreviations

AE: adverse event; ANA: anti-nuclear antibody; ANC: absolute neutrophil count; BlyS: B-lymphocyte stimulator; dsDNA: double-stranded DNA; ELISA: enzyme-linked immunosorbent assay; HAHA: human anti-human antibody; mAb: monoclonal antibody; PGA: Physician's Global Disease Assessment; SELENA: Safety of Estrogens in Lupus Erythematosus National Assessment; SF-36: 36-item Short Form; SLE: systemic lupus erythematosus; SLEDAI: Systemic Lupus Erythematosus Disease Activity Index; TNF: tumor necrosis factor.

## Competing interests

JZ, WC, and WF were all employees of Human Genome Sciences (HGS) at the time when the trial was conducted. RF, WS, EG, AW, JM, and WC declare research funding for this study provided by HGS. WS, AW, and JM declare they serve as consultants to HGS. WS also declares research support from HGS and Genentech, Inc., consulting to Genentech, and clinical trial support from Amgen Inc. MB, NM, and JM declare that they have no competing interests.

## Authors' contributions

RF, WS, EG, MB, NM, WC, JM, AW, and WM participated in the trial as principal investigators at their respective institutions. JZ was the lead biostatistician at Human Genome Sciences (HGS). WC was the lead pharmacokineticist at HGS. WF was the lead medical monitor at HGS. All authors contributed to the design of the trial. RF, WS, JZ, and WF drafted the manuscript.
